# GIP/MZT1 proteins orchestrate nuclear shaping

**DOI:** 10.3389/fpls.2014.00029

**Published:** 2014-02-07

**Authors:** Morgane Batzenschlager, Etienne Herzog, Guy Houlné, Anne-Catherine Schmit, Marie-Edith Chabouté

**Affiliations:** Institut de Biologie Moléculaire des Plantes, Centre National de la Recherche Scientifique, UPR 2357, Conventionné avec l’Université de StrasbourgStrasbourg, France

**Keywords:** nucleocytoplasmic continuum, gamma-tubulin complex, spindle assembly, nuclear envelope proteins, *Arabidopsis thaliana*

## Abstract

The functional organization of the nuclear envelope (NE) is only just emerging in plants with the recent characterization of NE protein complexes and their molecular links to the actin cytoskeleton. The NE also plays a role in microtubule nucleation by recruiting γ-Tubulin Complexes (γ-TuCs) which contribute to the establishment of a robust mitotic spindle. γ-tubulin Complex Protein 3 (GCP3)-interacting proteins (GIPs) have been identified recently as integral components of γ-TuCs. GIPs have been conserved throughout evolution and are also named MZT1 (mitotic-spindle organizing protein 1). This review focuses on recent data investigating the role of GIP/MZT1 at the NE, including insights from the study of GIP partners. It also uncovers new functions for GIP/MZT1 during interphase and highlights a current view of NE-associated components which are critical for nuclear shaping during both cell division and differentiation.

## INTRODUCTION

The nuclear envelope (NE) primarily appeared as a selective barrier between the nucleoplasm and the cytoplasm of Eukaryote cells. However, recent data revealed a rather complex interplay between both compartments, with the NE not only controlling fluxes through nuclear pore complexes (NPCs), but also participating in signal transduction cascades through the establishment of a nucleocytoplasmic continuum (Rothballer and Kutay, 2013). At its outside periphery, the NE allows the anchoring of cytoskeletal proteins and inside, the NE binds to the lamina and chromatin through specific integral membrane proteins. Bridging the inner (INM) and the outer (ONM) nuclear membranes, LInker of the Nucleoskeleton and the Cytoskeleton (LINC) complexes are considered as force transducers between the nuclear lamina and the cytoskeleton (Wang et al., 2012). LINC complexes have been characterized in animal cells (Rothballer and Kutay, 2013) and more recently in plants (Zhou and Meier, 2013). According to cell type specificity, NE composition and function diverge and appear to be critical during differentiation and development (Gomez-Cavazos and Hetzer, 2012).

The LINC complexes are mainly composed of Sad1/UNC84 domain proteins (SUNs) which are generally located at the INM (Tzur et al., 2006), interacting in the perinuclear space with Klarsicht/ANC-1/Syne (KASH) domain proteins anchored at the ONM. In animals, KASH domain proteins connect intermediate filaments, actin, microtubules (MTs), and/or motor proteins (dynein, kinesin). On the nucleoplasmic side, SUNs interact with lamins and/or lamin-associated proteins which constitute the lamina meshwork (Méjat and Misteli, 2010). Closely associated to the INM, the lamina contributes to the mechanical stability and elasticity of the nucleus (Funkhouser et al., 2013). Lamins interact with chromatin and play a role in transcriptional regulation, as well as genome and chromatin organization (Dechat et al., 2008). While SUN proteins have been evolutionary conserved in a remarkable way, KASH proteins have diverged in their sequence and function (Fridkin et al., 2009; Sosa et al., 2013). Altogether, animal LINC complexes are main actors in fundamental functions such as the determination of the NE structure and nuclear shaping, the organization of the cytoskeleton, force transmission at the NE, nuclear anchoring and movement, and genome organization, which leads to controlled gene expression programs (Razafsky and Hodzic, 2009; Starr and Fridolfsson, 2010; Rothballer and Kutay, 2013). It is therefore not surprising that any disruption of these connections causes diverse diseases. Nesprin (KASH domain protein), lamin and lamin-binding protein defects are symptomatic of envelopathies, including muscular dystrophies, cardiomyopathies, and premature aging. At the cellular level, these pathologies are characterized by a lobulation of the nucleus and modifications in the NE structure and its protein organization (Bonne and Quijano-Roy, 2013). It is thus essential – from both a medical and purely biological point of view – to understand how the nuclear shape is regulated and to identify the proteins which contribute to nuclear morphology.

In plant cells, similar nucleocytoplasmic crosstalks have been suspected for a long time, but genetic divergences throughout evolution in LINC complex genes and lamin counterparts delayed their characterization. Nevertheless, the association of several proteins with the nuclear membranes has been described. Among them, the Ran GTPase-activating protein RanGAP1 of *Arabidopsis* is targeted to the NE through its N-terminal, plant-specific WPP domain (Meier, 2000; Rose and Meier, 2001; Pay et al., 2002). WPP-domain interacting proteins (WIPs) are transmembrane proteins associated with the ONM (Xu et al., 2007), and are the only KASH-like members characterized so far (Meier and Brkljacic, 2009; Zhou et al., 2012). WIPs also interact with WPP domain-Interacting Tail-anchored proteins (WITs) which link the ONM to the actin cytoskeleton through MyosinXI-i (Tamura et al., 2013). SUN proteins are present (Graumann and Evans, 2013; Zhou and Meier, 2013) at the INM where a plant lamina meshwork, called plamina, does also exist (Fiserova et al., 2009; Goldberg, 2013). LITTLE NUCLEI (LINC)/Nuclear Matrix Constituent Proteins (NM) are located in the plamina (Masuda et al., 1997; Dittmer et al., 2007; Kimura et al., 2010), and are good candidates for functional homologues of animal lamins (Ciska and Moreno Diaz de la Espina, 2013; Graumann and Evans, 2013; Sakamoto and Takagi, 2013). However, the connections between INM proteins or plamina components and chromatin have not been described so far.

In the various Eukaryotic cell types, the nuclear surface plays a central role in establishing fully functional MT nucleation and organizing sites. MTs are nucleated from gamma-Tubulin Complexes (γ-TuCs) which are concentrated at the centrosome, positioned close to the nucleus in animal cells, at spindle pole bodies (SPBs) in yeast, or dispersed at various MT nucleating sites located at the plasma membrane, on preexisting MTs and at the NE in plant cells. On the cytoplasmic side, direct links between γ-TuCs and the NE remain poorly understood. *Caenorhabditis elegans* KASH domain proteins have been reported to participate in centrosome attachment to the NE (Minn et al., 2009) and in *Schizosaccharomyces pombe,* they link the NE to the SPB where MTs are nucleated (King et al., 2008). In parallel, a novel concept of NE shaping linked to chromatin-bound MT regulation is only just emerging. Developmental pluripotency-associated 2 (Dppa2), a chromatin-binding factor, regulates NE assembly in *Xenopus *egg extracts through temporally and spatially restricted MT polymerization (Xue et al., 2013). MT dynamics also induce NE and chromatin fiber reshaping in mouse melanoma cells (Gerlitz et al., 2013). In plants, the recent discovery of GIP proteins (Janski et al., 2008, 2012, Nakamura et al., 2012) as integral components of the γ-TuC and the GIP’s partnership (Batzenschlager et al., 2013) tend to ascribe GIP a major role in NE shaping in both cycling and differentiated cells. This review will summarize our current view of a plant nucleocytoplasmic continuum based on genetic and cellular analyses.

## ROLE OF GIPs AT THE ONM IN γ-TuC RECRUITMENT AND ANCHORING

In plants, the whole nuclear surface has been characterized as a MT nucleating site (Erhardt et al., 2002; Seltzer et al., 2003; Canaday et al., 2005; Seltzer et al., 2007); perinuclear MT arrays may connect the NE to the cellular periphery and participate in signal transduction. The γ-tubulin complex proteins GCP2 and GCP3 have been detected at the nuclear periphery (Seltzer et al., 2007). However, no transmembrane domain has been identified within the GCP sequences, indicating that the association of GCPs with the NE requires specific anchoring proteins. In order to identify such components, GCP3 was used as a bait in a yeast two-hybrid screen which allowed the identification of GCP3 Interacting Proteins (GIPs; Janski et al., 2008, 2012). GIPs are small proteins of about 8 kDa encoded by two gene copies in plants. These proteins are mainly structured in three alpha helices (Batzenschlager et al., 2013). GIP1 or GIP2 complemented *gip1gip2* knock down mutants at the organism level (Janski et al., 2012), suggesting that both genes mostly shared redundant functions. GCP localization studies performed in *gip* knock down mutants revealed the role of GIPs in γ-TuC recruitment at the NE and mitotic MT arrays (Janski et al., 2012). In *S. pombe*, while the GIP homologue Mzt1/Tam4 was dispensable for the assembly of the γ-TuC core, it was required for its recruitment at the SPB (Dhani et al., 2013; Masuda et al., 2013). Therefore, considering their strong conservation throughout evolution, GIPs appear as new actors regulating γ-TuC attachment at MT nucleation sites.

A further yeast two-hybrid screen of an *Arabidopsis* library, using GIP1 as a bait, led to the identification of TSA1 (At1g52410; Batzenschlager et al., 2013) – earlier described as TonSoku (TSK)-Associating protein 1 (Suzuki et al., 2005). TSA1 is an 84 kDa protein possessing an acidic repeat sequence, rich in alpha helices and coiled-coil domains, and able to mediate the multimerization of the protein. TSA1 also possesses a putative transmembrane domain (Suzuki et al., 2005) and is located, like GIPs, at the NE (Batzenschlager et al., 2013), suggesting its role in anchoring γ-TuCs at the ONM via a GCP3–GIP interaction.

## GIP FUNCTIONS ON BOTH SIDES OF THE NE IN *Arabidopsis*

Confocal observations of the NE region showed that chromocentres, GIPs and MTs were co-aligned (Batzenschlager et al., 2013). At the electron microscopy resolution, GIPs were localized on both sides of the NE and associated with heterochromatin. In addition, *gip1gip2* knock down mutants exhibited strongly affected nuclear morphology phenotypes (Janski et al., 2012; Batzenschlager et al., 2013). The flow cytometry analysis of cell DNA contents in these mutants showed that the depletion of GIP proteins led to a significant increase in ploidy levels. This was confirmed by the observation of highly enlarged nuclei in all the tissues analyzed, whether undifferentiated or differentiated. Moreover, the nucleus became lobulated, resembling the phenotypes observed in laminopathies (Mattout et al., 2006). The distribution of NPCs was also affected in *gip* mutants, showing an increase in NPC density and an NPC misshaping. Simultaneously, the distribution of AtSUN1 became heterogeneous, suggesting a severe modification of the NE organization (Batzenschlager et al., 2013). These nuclear phenotypes are the strongest ever observed in mutated plant cells, suggesting that GIPs play a key role in nuclear shaping and organization, in addition to their role during mitosis (Janski et al., 2012).

**FIGURE 1:**
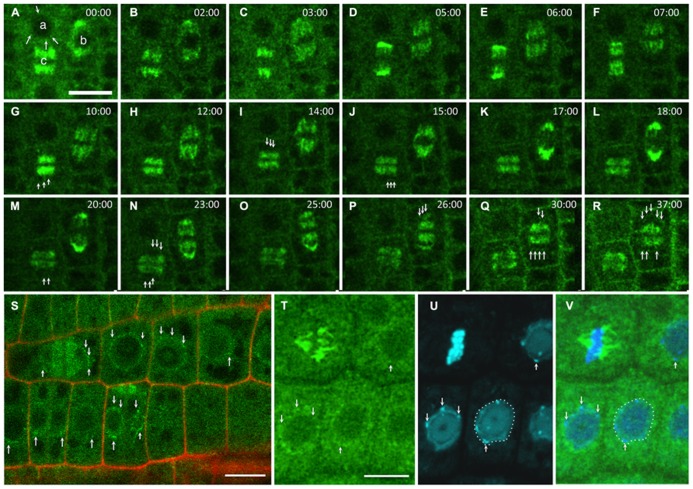
**GIP dynamics throughout the cell cycle.** Time-lapse images of the expression of pGIP1::AtGIP1-GFP in an *Arabidopsis* root tip. Three cells were followed for 37 min, using confocal microscopy **(A–R)**. **(A)** GIP1 localizes in a dotted pattern (arrows) at the nuclear periphery of an interphase cell (a), on a prophase spindle (b) and on a metaphase spindle (c). In end-anaphase-telophase transition, GIP1 remains associated with remnant kinetochore fibers and redistributes in a dotted pattern to the newly built NE (arrows in **G, I, J, M** and **N** of the (c) cell and in **P–R** of the (b) cell). **(S)** Larger view of a GIP1-GFP expressing root tip in which the nuclear periphery is labeled in interphase and telophase cells (arrows). (**T–V**) DAPI staining of GIP1–GFP cells confirming that GIP is located at the nuclear periphery. The arrows indicate that some GIP1–GFP signals are located close to chromocentres which are under the inner nuclear membrane. Bar = 10 μm.

Ploidy abnormalities may be the consequence of defects in replication and/or genome maintenance after NE reformation at the chromatin level or in mitosis regulation at the MT level. GIPs are present at the NE during interphase (**Figure 1A**, arrows), on spindle fibers during mitosis (**Figures 1A–Q**) and on the reforming NE of daughter nuclei (**Figures 1G,I,J,M,N,Q,R**, arrows). *gip1gip2* mutants show either unstable spindles associated with lagging chromosomes and micronuclei formation or metaphase arrest and polyploid restitution nuclei which may explain both aneu- and polyploid resulting cells (Janski et al., 2012). Similarly, MOZART1, the human GIP homologue (Hutchins et al., 2010; Teixidó-Travesa et al., 2010), is a γ-TuC component whose depletion leads to the accumulation of prometaphase cells with monopolar spindles and closely spaced centrosome pairs (Hutchins et al., 2010), as observed for γ-tubulin (TUBG1) depletion. *S. pombe* temperature-sensitive or deletion *mzt1/Tam4* mutants display defects in MT organization during both interphase and mitosis, showing abnormal anaphases and cytokinesis defects (Dhani et al., 2013; Masuda et al., 2013). These phenotypes suggest a common function of GIPs/MZT1 in mitotic regulation. Furthermore, NE proteins remain present at the spindle matrix during division. Indeed, the INM proteins SUN1 and SUN2 were found in the spindle and close to the spindle poles of tobacco BY-2 and *Arabidopsis* cells (Graumann and Evans, 2011; Oda and Fukuda, 2011). A localization in the close proximity of chromatin was suggested for SUNs throughout division, but a SUN/chromatin complex has not been identified in plants. Interestingly, TSA1 was also found at the mitotic spindle poles where its partner, the chromatin-remodeling factor TSK, is accumulated (Suzuki et al., 2005). A similar pattern of GIP accumulation was observed within the spindle and at poles in telophase, with GIP1 harboring a punctuated distribution (**Figure 1**, arrows). This suggests a close connection between GIP and chromatin during NE reformation (Batzenschlager et al., 2013), which may be linked to the nuclear function of TSK in chromatin organization and epigenetic gene silencing (Takeda et al., 2004; Ohno et al., 2011). Ploidy may be controlled at the chromatin level by the coordinated activity of proteins which ensure controlled gene expression and replication, chromatin remodeling and chromosome organization. The presence of GIPs in heterochromatin suggests a role of these proteins in such processes. Unraveling the molecular and genetic relationships between GIP, TSA1 and TSK may clarify this functional aspect.

Interestingly, the NPC biogenesis is altered in *gip* mutants which exhibit misshaped NPCs (Batzenschlager et al., 2013). Whether the function of GIP in telophase is required for NPC assembly and NE reformation, which are tightly coordinated processes in metazoans (Güttinger et al., 2009), remains to be determined.

## NUCLEAR SHAPE PHENOTYPES IN DIFFERENTIATED/UNDIFFERENTIATED CELLS OF *Arabidopsis* MUTANTS

In cells lacking the major LINC components characterized so far, defects in the localization of NE-associated proteins and nuclear shaping were often restricted either to the cycling or the differentiated states. Indeed, *wip1-1 wip2-1 wip3-1* triple mutants lost the accumulation of RanGAP1 at the NE only in undifferentiated root tip cells of *Arabidopsis* but did not show peculiar plant growth or NE shape defects. RanGAP1 was also delocalized from the NE in *wit1 wit2 *double mutant root tips.

Regarding the nuclear shape, the nuclei of trichomes, leaf epidermal cells and mature root hair cells of *wip1-1 wip2-1 wip3-1* plants showed a mild change from an elongated to a more roundish form (Zhou et al., 2012), suggesting that other proteins were involved in the control of the nuclear shape. Similarly to *wip* mutants, the *sun1-KO sun2-KD* mutant did not show major defects in nuclear positioning or plant development and the nuclei of differentiated cells only became less elongated (Oda and Fukuda, 2011; Zhou et al., 2012). This suggests that defects in plant SUN or KASH domain proteins are less harmful than in their animal SUN-KASH domain counterparts. Concerning plamina components, *nmcp1/linc1* and* nmcp2/linc2* mutants exhibited a reduced nuclear volume with increasing density of DNA packaging in nuclei of differentiated tissues. However, when expressed under its own promoter, LINC1-GFP preferentially labeled meristematic cell nuclei, but not differentiated root cell nuclei (Dittmer and Richards, 2008). This suggests that in meristematic tissues other NE components may compensate for defects in LINCs/NMCPs for nuclear shaping.

Interestingly, the variations of nucleus morphology described for the *wip*, *sun,* and *nmcp *mutants were**observed in differentiated cells (Zhou et al., 2012; Ciska and Moreno Diaz de la Espina, 2013). On the contrary, the dramatic lobulation of *gip *nuclei was observed in both cycling (root tip) and differentiated cells (cotyledons, leaves and petals; Batzenschlager et al., 2013), suggesting that GIPs were key components involved in the nuclear shape of somatic cells all along the development. Their location at both NE sides and their ability to multimerize are in agreement with (i) their role in γ-TuC recruitment and the regulation of MT array organization and (ii) their interaction with a TSA1–TSK complex, participating in chromatin organization through a nucleocytoplasmic continuum.

## WORKING MODEL FOR GIP FUNCTIONS AT THE NE

**FIGURE 2:**
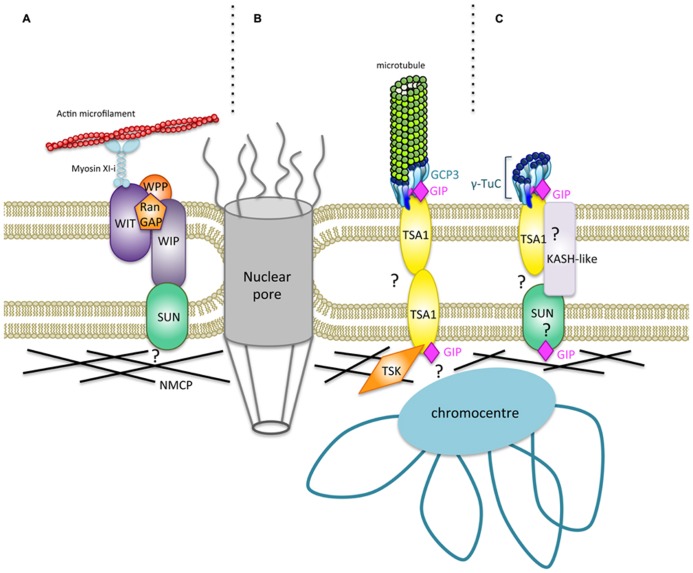
**Hypothetical model for a new type of nucleocytoplasmic bridges regulating the shape of *Arabidopsis thaliana* nuclei.** **(A)** A specific plant LINC complex has been characterized, and it plays a role in nuclear movement and shaping (Meier and Brkljacic, 2009; Graumann and Evans, 2010; Zhou et al., 2012; Tamura et al., 2013). Trimeric organization of SUN domain proteins (Sosa et al., 2012) has been omitted for more clarity. A WIT–WIP complex interacts with WWP proteins and RanGAP. WIT also interacts with Myosin XI-i which links the actin cytoskeleton to the ONM. The WIP–SUN complex constitutes the first core LINC identified in plants. NMCPs/LINCs are plamina components (Ciska and Moreno Diaz de la Espina, 2013) which may interact with SUN (Graumann et al., 2013). **(B)** A new nucleocytoplasmic continuum may consist of GIPs which bind both GCP3, a component of the γ-TuC involved in MT nucleation at the NE, and TSA1, a putative transmembrane protein. TSK localizes in the nucleoplasm (Suzuki et al., 2004; Takeda et al., 2004; Ohno et al., 2011) and may associate with TSA1 at the INM. Both TSK and GIP binding domains of TSA1 are overlapping at its C-terminus. TSA1 possesses coiled-coil domains which may allow multimerization of the protein. Multimerization propensity of GIP/MZT1 proteins have also been reported and could reinforce the interaction with their partners (Batzenschlager et al., 2013; Dhani et al., 2013). Such a model may at least exist in cycling cells. **(C)** The continuum may also involve a subpopulation of γ-TuCs which does not nucleate MTs in differentiated cells. The GIP–TSA1 complex may interact with a still unknown KASH-like protein associated to SUN in the perinuclear space. The molecular interplay at the INM, plamina and chromatin interface needs to be further characterized.

The identification of LINC complex components is emerging in higher plants. At least two types of WIP–SUN complexes are present in *Arabidopsis* – one which is involved in nuclear shape and movement, including myosin XI-i and WITs (Tamura et al., 2013), and another with RanGAP or WPP proteins for nucleo-cytoplasmic trafficking (Zhou and Meier, 2013; **Figure 2A**). In addition, other protein complexes may coexist at the NE interface. The model presented in **Figures 2B,C** illustrates the molecular interplay between GIPs and their identified and possible partners at the NE. WIPs possess a C-terminal PNS tail which terminates in a ϕ-VPT motif (ϕ, hydrophobic amino acid) which shares partial similarity with the PPPX motif of animal KASH domain proteins (Xu et al., 2007). These motifs are essential for interaction with SUN and NE localization of WIP and KASH proteins (Razafsky and Hodzic, 2009; Starr and Fridolfsson, 2010; Zhou et al., 2012). Interestingly, the GIP interacting protein TSA1 harbors a transmembrane domain close to a VIPT motif and is located at the NE (Batzenschlager et al., 2013). Therefore, TSA1 constitutes an attractive candidate for γ-TuC anchoring at the NE in *Arabidopsis*. The presence of several coiled- coil domains in TSA1 may favor its multimerization as is the case for SUN, KASH (Sosa et al., 2012) and GIP proteins (Batzenschlager et al., 2013). Such multimers may occur between TSA1 or involve additional NE proteins in the perinuclear space. TSA1–TSA1 or TSA1–KASH–SUN complexes may constitute structured platforms, helping γ-TuC attachment and organization. On the nuclear side, LINCs/NMCPs are coiled-coil proteins conserved in multicellular plants (Ciska and Moreno Diaz de la Espina, 2013) and they may interact with SUN proteins (Graumann et al., 2013). Since TSA1 was also shown to interact with the chromatin remodeling factor TSK (Suzuki et al., 2005), TSA1 can possibly locate at both the ONM and the INM. Therefore, a GIP–TSA1 interaction could also occur at the INM interface.

The loss of the continuum integrity should disturb the forces applied at the NE – originating either from the cytoskeleton and/or the nucleoskeleton – and result in nuclear deformations, as observed in animal cells. This was remarkably observed when *GIP* genes were mutated in *Arabidopsis*, and it was probably due to the absence of GIPs on both NE sides. GIP localization studies indicate that GIPs may be involved in molecular links between MT nucleation sites at the ONM and chromocentres at the INM. This is in agreement with the intranuclear localization of GIPs and their possible roles in the intranuclear environment. It may be possible that GIPs regulate chromatin organization and/or gene expression. The phenotypic pleiotropy observed in *Arabidopsis* *gip1gip2* mutants may indicate that these proteins play active roles during the cell cycle for γ-TuC recruitment in close connection with chromatin organization (Batzenschlager et al., 2013).

In the proposed model, γ-TuC recruitment is either linked to MT nucleation (**Figure 2B**) or does not imply MT assembly. In the latter case, we can consider the existence of a subpopulation of non-nucleating γ-TuCs at the NE (**Figure 2C**). The presence of such specific γ-TuCs may be linked to differentiation, a status in which nuclei are along a cell edge and perinuclear MT nucleation is switched off. This is in agreement with the absence of nuclear shape alteration in differentiated cells after using MT depolymerization drugs (Sakamoto and Takagi, 2013).

Altogether, considering established and suspected GIP functions, further studies about these small proteins could be of particular interest to decipher physical connections and signaling at the nucleocytoplasmic continuum in plant and other Eukaryotic cells.

## Conflict of Interest Statement

The authors declare that the research was conducted in the absence of any commercial or financial relationships that could be construed as a potential conflict of interest.

## References

[B1] Batzenschlager M. Masoud K. Janski N. Houlné G. Herzog E. Evrard J.-L. (2013). The GIP gamma-tubulin complex-associated proteins are involved in nuclear architecture in *Arabidopsis thaliana*. *Front. Plant Sci*. 4:480 10.3389/fpls.2013.00480 PMC3842039 24348487

[B2] Bonne G. Quijano-Roy S. (2013). Emery-dreifuss muscular dystrophy, laminopathies, and other nuclear envelopathies. *Handb. Clin. Neurol.* 113 1367–1376 10.1016/B978-0-444-59565-2.00007-1 23622360

[B3] Canaday J. Brochot A. L. Seltzer V. Herzog E. Evrard J. L. Schmit A. C. (2005) “Microtubule assembly in higher plants,” in *Recent Research Developments in Molecular Biology* Vol. 2 ed. Pandalai S. G. (Trivandrum, India: Research Signpost) 103–119

[B4] Ciska M Moreno Diaz de la Espina S. (2013). NMCP/LINC proteins: putative lamin analogs in plants? *Plant Signal. Behav*. 8, pii: e26669. 10.4161/psb.26669 PMC4091594 24128696

[B5] Dechat T. Pfleghaar K. Sengupta K. Shimi T. Shumaker D. Solimando L. (2008). Nuclear lamins: major factors in the structural organization and function of the nucleus and chromatin. *Genes Dev.* 22 832–853 10.1101/gad.1652708 18381888 PMC2732390

[B6] Dhani D. Goult B. George G. Rogerson D. Bitton D. Miller C. (2013). Mzt1/Tam4, a fission yeast MOZART1 homologue, is an essential component of the γ-tubulin complex and directly interacts with GCP3Alp6. *Mol. Biol. Cell* 24 3337–3349 10.1091/mbc.E13-05-0253 24006493 PMC3814152

[B7] Dittmer T. Richards E. (2008). Role of LINC proteins in plant nuclear morphology. *Plant Signal. Behav.* 3 485–487 10.4161/psb.3.7.5682 19704494 PMC2634438

[B8] Dittmer T. Stacey N. Sugimoto-Shirasu K. Richards E. (2007). LITTLE NUCLEI genes affecting nuclear morphology in *Arabidopsis thaliana*. *Plant Cell* 19 2793–2803 10.1105/tpc.107.053231 17873096 PMC2048703

[B9] Erhardt M. Stoppin-Mellet V. Campagne S. Canaday J. Mutterer J. Fabian T. (2002). The plant Spc98p homologue colocalizes with gamma-tubulin at microtubule nucleation sites and is required for microtubule nucleation. *J. Cell Sci.* 115 2423–2431 12006626 10.1242/jcs.115.11.2423

[B10] Fiserova J. Kiseleva E. Goldberg M. (2009). Nuclear envelope and nuclear pore complex structure and organization in tobacco BY-2 cells. *Plant J.* 59 243–255 10.1111/j.1365-313X.2009.03865.x 19392704

[B11] Fridkin A. Penkner A. Jantsch V. Gruenbaum Y. (2009). SUN-domain and KASH-domain proteins during development, meiosis and disease. *Cell. Mol. Life Sci.* 66 1518–1533 10.1007/s00018-008-8713-y 19125221 PMC6485414

[B12] Funkhouser C. Sknepnek R. Shimi T. Goldman A. Goldman R Olvera de la Cruz M. (2013). Mechanical model of blebbing in nuclear lamin meshworks. *Proc. Natl. Acad. Sci. U.S.A.* 110 3248–3253 10.1073/pnas.1300215110 23401537 PMC3587257

[B13] Gerlitz G. Reiner O. Bustin M. (2013). Microtubule dynamics alter the interphase nucleus. *Cell. Mol. Life Sci.* 70 1255–1268 10.1007/s00018-012-1200-5 23117601 PMC11113956

[B14] Goldberg M. W. (2013). Nucleoskeleton in plants: the functional organization of filaments in the nucleus. *Ann. Plant Rev*. 46 93–122 ch4 10.1002/9781118472507.ch4

[B15] Gomez-Cavazos J. Hetzer M. (2012). Outfits for different occasions: tissue-specific roles of Nuclear Envelope proteins. *Curr. Opin. Cell Biol.* 24 775–783 10.1016/j.ceb.2012.08.008 22995343 PMC3587307

[B16] Graumann K. Bass H. Parry G. (2013). SUNrises on the International Plant Nucleus Consortium: SEB Salzburg 2012. *Nucleus* 4 3–7 10.4161/nucl.23385 23324458 PMC3585025

[B17] Graumann K. Evans D. (2010). Plant SUN domain proteins: components of putative plant LINC complexes? *Plant Signal Behav*. 5 154–156 10.4161/psb.5.2.10458 20023391 PMC2884122

[B18] Graumann K. Evans D. (2011). Nuclear envelope dynamics during plant cell division suggest common mechanisms between kingdoms. *Biochem. J.* 435 661–667 10.1042/BJ20101769 21323637

[B19] Graumann K. Evans D (2013). The nuclear envelope – structure and protein interactions. *Ann. Plant Rev.* 46 19–55 ch2 10.1002/9781118472507.ch2

[B20] Güttinger S. Laurell E. Kutay U. (2009). Orchestrating nuclear envelope disassembly and reassembly during mitosis. *Nature Rev. Mol. Cell. Biol.* 10 178–191 10.1038/nrm2641 19234477

[B21] Hutchins J. Toyoda Y. Hegemann B. Poser I. Hériché J.-K. Sykora M. (2010). Systematic analysis of human protein complexes identifies chromosome segregation proteins. *Science* 328 593–599 10.1126/science.1181348 20360068 PMC2989461

[B22] Janski N. Herzog E. Schmit A.-C. (2008). Identification of a novel small *Arabidopsis* protein interacting with gamma-tubulin complex protein 3. *Cell Biol. Int.* 32 546–548 10.1016/j.cellbi.2007.11.006 18178112

[B23] Janski N. Masoud K. Batzenschlager M. Herzog E. Evrard J.-L. Houlné G. (2012). The GCP3-interacting proteins GIP1 and GIP2 are required for γ-tubulin complex protein localization, spindle integrity, and chromosomal stability. *Plant Cell* 24 1171–1187 10.1105/tpc.111.094904 22427335 PMC3336128

[B24] Kimura Y. Kuroda C. Masuda K. (2010). Differential nuclear envelope assembly at the end of mitosis in suspension-cultured Apium graveolens cells. *Chromosoma* 119 195–204 10.1007/s00412-009-0248-y 19997923

[B25] King M. Drivas T. Blobel G. (2008). A network of nuclear envelope membrane proteins linking centromeres to microtubules. *Cell* 134 427–438 10.1016/j.cell.2008.06.022 18692466 PMC2617791

[B26] Masuda H. Mori R. Yukawa M. Toda T. (2013). Fission yeast MOZART1/Mzt1 is an essential (γ -tubulin complex component required for complex recruitment to the microtubule organizing center, but not its assembly. *Mol. Biol. Cell* 24 2894–2906 10.1091/mbc.E13-05-0235 23885124 PMC3771951

[B27] Masuda K. Xu Z. Takahashi S. Ito A. Ono M. Nomura K. (1997). Peripheral framework of carrot cell nucleus contains a novel protein predicted to exhibit a long alpha-helical domain. *Exp. Cell. Res.* 232 173–181 10.1006/excr.1997.3531 9141634

[B28] Mattout A. Dechat T. Adam S. A. Goldman R. D. Gruenbaum Y. (2006). Nuclear lamins, diseases and aging. *Curr. Opin. Cell Biol.* 18 335–341 10.1016/j.ceb.2006.03.007 16632339

[B29] Meier I. (2000). A novel link between ran signal transduction and nuclear envelope proteins in plants. *Plant Physiol.* 124 1507–1510 10.1104/pp.124.4.1507 11115866 PMC1539304

[B30] Meier I. Brkljacic J. (2009). Adding pieces to the puzzling plant nuclear envelope. *Curr. Opin. Plant Biol.* 12 752–759 10.1016/j.pbi.2009.09.016 19875325

[B31] Méjat A. Misteli T. (2010). LINC complexes in health and disease. *Nucleus* 1 40–52 10.4161/nucl.1.1.10530 21327104 PMC3035119

[B32] Minn I. Rolls M. Hanna-Rose W. Malone C. (2009). SUN-1 and ZYG-12, mediators of centrosome-nucleus attachment, are a functional SUN/KASH pair in *Caenorhabditis elegans*. *Mol. Biol. Cell* 20 4586–4595 10.1091/mbc.E08-10-1034 19759181 PMC2770946

[B33] Nakamura M. Yagi N. Kato T. Fujita S. Kawashima N. Ehrhardt D. (2012). *Arabidopsis* GCP3-interacting protein 1/MOZART 1 is an integral component of the γ-tubulin-containing microtubule nucleating complex. *Plant J.* 71 216–225 10.1111/j.1365-313X.2012.04988.x 22404201

[B34] Oda Y. Fukuda H. (2011). Dynamics of *Arabidopsis* SUN proteins during mitosis and their involvement in nuclear shaping. *Plant J.* 66 629–641 10.1111/j.1365-313X.2011.04523.x 21294795

[B35] Ohno Y. Narangajavana J. Yamamoto A. Hattori T. Kagaya Y. Paszkowski J. (2011). Ectopic gene expression and organogenesis in *Arabidopsis* mutants missing BRU1 required for genome maintenance. *Genetics* 189 83–95 10.1534/genetics.111.130062 21705754 PMC3176131

[B36] Pay A. Resch K. Frohnmeyer H. Fejes E. Nagy F. Nick P. (2002). Plant RanGAPs are localized at the nuclear envelope in interphase and associated with microtubules in mitotic cells. *Plant J.* 30 699–709 10.1046/j.1365-313X.2002.01324.x 12061901

[B37] Razafsky D. Hodzic D. (2009). Bringing KASH under the SUN: the many faces of nucleo-cytoskeletal connections. *J. Cell Biol.* 186 461–472 10.1083/jcb.200906068 19687252 PMC2733748

[B38] Rose A. Meier I. (2001). A domain unique to plant RanGAP is responsible for its targeting to the plant nuclear rim. *Proc. Natl. Acad. Sci. U.S.A* 98 15377–15382 10.1073/pnas.261459698 11752475 PMC65037

[B39] Rothballer A. Kutay U. (2013). Poring over pores: nuclear pore complex insertion into the nuclear envelope. *Trends Biochem. Sci.* 38 292–301 10.1016/j.tibs.2013.04.001 23639636

[B40] Sakamoto Y. Takagi S. (2013). LITTLE NUCLEI 1 and 4 regulate nuclear morphology in *Arabidopsis thaliana*. *Plant Cell Phys.* 54 622–633 10.1093/pcp/pct031 23396599

[B41] Seltzer V. Janski N. Canaday J. Herzog E. Erhardt M. Evrard J.-L. (2007). *Arabidopsis* GCP2 and GCP3 are part of a soluble gamma-tubulin complex and have nuclear envelope targeting domains. *Plant J.* 52 322–331 10.1111/j.1365-313X.2007.03240.x 17714428

[B42] Seltzer V. Pawlowski T. Campagne S. Canaday J. Erhardt M. Evrard J.-L. (2003). Multiple microtubule nucleation sites in higher plants. *Cell Biol. Int.* 27 267–269 10.1016/S1065-6995(02)00345-1 12681331

[B43] Sosa B. Kutay U. Schwartz T. (2013). Structural insights into LINC complexes. *Curr. Opin. Struct. Biol.* 23 285–291 10.1016/j.sbi.2013.03.005 23597672 PMC4077334

[B44] Sosa B. Rothballer A. Kutay U. Schwartz T. (2012). LINC complexes form by binding of three KASH peptides to domain interfaces of trimeric SUN proteins. *Cell* 149 1035–1047 10.1016/j.cell.2012.03.046 22632968 PMC3383001

[B45] Starr D. Fridolfsson H. (2010). Interactions between nuclei and the cytoskeleton are mediated by SUN-KASH nuclear-envelope bridges. *Annu. Rev. Cell. Dev. Biol.* 26 421–444 10.1146/annurev-cellbio-100109-104037 20507227 PMC4053175

[B46] Suzuki T. Inagaki S. Nakajima S. Akashi T. Ohto M.-A. Kobayashi M. (2004). A novel *Arabidopsis* gene TONSOKU is required for proper cell arrangement in root and shoot apical meristems. *Plant J.* 38 673–684 10.1111/j.1365-313X.2004.02074.x 15125773

[B47] Suzuki T. Nakajima S. Inagaki S. Hirano-Nakakita M. Matsuoka K. Demura T. (2005). TONSOKU is expressed in S phase of the cell cycle and its defect delays cell cycle progression in *Arabidopsis*. *Plant Cell Physiol.* 46 736–742 10.1093/pcp/pci082 15746155

[B48] Takeda S. Tadele Z. Hofmann I. Probst A. V. Angelis K. J. Kaya H. (2004). BRU1, a novel link between responses to DNA damage and epigenetic gene silencing in *Arabidopsis*. *Genes Dev.* 18 782–793 10.1101/gad.295404 15082530 PMC387418

[B49] Tamura K. Iwabuchi K. Fukao Y. Kondo M. Okamoto K. Ueda H. (2013). Myosin XI-i links the nuclear membrane to the cytoskeleton to control nuclear movement and shape in *Arabidopsis*. *Curr. Biol.* 23 1776–1781 10.1016/j.cub.2013.07.035 23973298

[B50] Teixidó-Travesa N. Villen J. Lacasa C. Bertran M. T. Archinti M. A. Gygi C. (2010). The gammaTuRC revisited: a comparative analysis of interphase and mitotic human gamma TuRC redefines the set of core components and identifies the novel subunit GCP8. *Mol. Biol. Cell* 21 3963–3972 10.1091/mbc.E10-05-0408 20861304 PMC2982109

[B51] Tzur Y. Wilson K. Gruenbaum Y. (2006). SUN-domain proteins: “Velcro” that links the nucleoskeleton to the cytoskeleton. *Nat. Rev. Mol. Cell Biol.* 7 782–788 10.1038/nrm2003 16926857

[B52] Wang W. Shi Z. Jiao S. Chen C. Wang H. Liu G. (2012). Structural insights into SUN-KASH complexes across the nuclear envelope. *Cell Res.* 22 1440–1452 10.1038/cr.2012.126 22945352 PMC3463262

[B53] Xu X. M. Meulia T. Meier I. (2007). Anchorage of plant RanGAP to the nuclear envelope involves novel nuclear-pore-associated proteins. *Curr. Biol.* 17 1157–1163 10.1016/j.cub.2007.05.076 17600715

[B54] Xue J. Woo E. Postow L. Chait B. Funabiki H. (2013). Chromatin-bound Xenopus dppa2 shapes the nucleus by locally inhibiting microtubule assembly. *Dev. Cell* 27 47–59 10.1016/j.devcel.2013.08.002 24075807 PMC3800493

[B55] Zhou X. Graumann K. Evans D. Meier I. (2012). Novel plant SUN-KASH bridges are involved in RanGAP anchoring and nuclear shape determination. *J. Cell Biol*. 196 203–211 10.1083/jcb.201108098 22270916 PMC3265956

[B56] Zhou X. Meier I. (2013). How plants LINC the SUN to KASH. *Nucleus* 4 206–215 10.4161/nucl.24088 23680964 PMC3720751

